# A Blueprint of Microstructures and Stage-Specific Transcriptome Dynamics of Cuticle Formation in *Bombyx mori*

**DOI:** 10.3390/ijms23095155

**Published:** 2022-05-05

**Authors:** Zhengwen Yan, Xiaoling Tong, Gao Xiong, Weike Yang, Kunpeng Lu, Yajie Yuan, Minjin Han, Hai Hu, Wei Wei, Fangyin Dai

**Affiliations:** 1 State Key Laboratory of Silkworm Genome Biology, Key Laboratory of Sericultural Biology and Genetic Breeding, Ministry of Agriculture and Rural Affairs, College of Sericulture, Textile and Biomass Sciences, Southwest University, Chongqing 400715, China; yanzhengwen@126.com (Z.Y.); xltong@swu.edu.cn (X.T.); xgao13@163.com (G.X.); wksun1985@163.com (W.Y.); lukunpeng@swu.edu.cn (K.L.); yuanyajie1@email.swu.edu.cn (Y.Y.); minjinhan@126.com (M.H.); huhaiswu@163.com (H.H.); 2 College of Notoginseng Medicine and Pharmacy, Wenshan University, Wenshan 663000, China; 3 The Sericultural and Apicultural Research Institute, Yunnan Academy of Agricultural Sciences, Honghe 661100, China; 4 Guangxi Academy of Sericultural Sciences, Nanning 530007, China; gxcanyeweiwei@126.com

**Keywords:** insect cuticle, cuticle structure, transcriptome analysis, gene family identification, protein components, knockout

## Abstract

Insect cuticle is critical for the environmental adaptability and insecticide resistance of insects. However, there is no clear understanding of the structure and protein components of the cuticle during each developmental stage of holometabolous insects, and knowledge about the protein components within each layer is vague. We conducted serial sectioning, cuticular structure analysis, and transcriptome sequencing of the larval, pupal, and adult cuticles of *Bombyx mori*. The deposition processes of epicuticle, exocuticle, and endocuticle during larval, pupal, and adult cuticle formation were similar. Transcriptome analysis showed that these cuticle formations share 74% of the expressed cuticular protein (CP) genes and 20 other structural protein genes, such as *larval serum protein* and *prisilkin*. There are seven, six, and eleven stage-specific expressed CP genes in larval, pupal, and adult cuticles, respectively. The types and levels of CP genes may be the key determinants of the properties of each cuticular layer. For example, the CPs of the RR-2 protein family with high contents of histidine (His) are more essential for the exocuticle. Functional analysis suggested that BmorCPAP1-H is involved in cuticle formation. This study not only offers an in-depth understanding of cuticle morphology and protein components but also facilitates the elucidation of molecular mechanisms underlying cuticle formation in future studies.

## 1. Introduction

Insects are one of the largest and most successful groups in the animal kingdom, and their success mainly depends on their strong adaptability to different environments. The insect cuticle is critical for the environmental adaptability of insects [1,2]. The cuticle covers the exterior of the insect body and functions as a protective barrier. It protects insects from environmental stresses, such as dehydration, pathogen invasion, insecticide penetration, and ultraviolet irradiation. The cuticle also functions as an attachment site for internal muscles and organs and it is a key component of locomotion and flight [3,4]. These features of the cuticle help insects, including pests, to adapt to complex environments. Moreover, reduced insecticide penetration across the cuticle is one of the main mechanisms of insecticide resistance in pests [5].

Cuticle formation has been studied from multiple aspects, including structure, composition, formation process, and the expression pattern of cuticle-related genes. The cuticle is a lamellar structure secreted by epidermal cells, and, from outside to inside, it is divided into three major layers. These include the epicuticle, exocuticle, and endocuticle [3,6]. The epicuticle lacks chitin and mainly contains sclerotized proteins impregnated with lipoproteins, lipids, and waxes. The exocuticle and endocuticle mainly contain chitin and cuticular proteins (CPs) [6,7]. To allow for growth and development, the cuticle is remolded during each molt. When the cuticle is remolding, three major layers are deposited successively, in the order of epicuticle, exocuticle, and endocuticle [8]. Cuticle-related genes are expressed before or after each molt, which is regulated by ecdysteroids and juvenile hormones [8]. The timing of expression defines the kind of CP that constructs the cuticular layer [1]. The CP genes involved in exocuticle and endocuticle formations are expressed before and after ecdysis, respectively [9].

The insect cuticle differs substantially in appearance and properties among larval, pupal, and adult stages. In the silkworm *Bombyx mori*, the colors of larval, pupal, and adult cuticles differ, and the adult cuticle is covered with scales. The differences in appearance and properties of larval, pupal, and adult cuticles depend on cuticle structures and protein components, and protein components are the structural basis of cuticle. Some protein components that are involved in the cuticle depositions of specific periods and layers have been reported in insects. For example, in *B. mori*, the BmorCPH24 is deposited in the larval endocuticle and the BmorCPH24 defective mutant larva is more sensitive to environmental stresses [2,10]. In the mosquito *Anopheles gambiae*, immunolocalization of one RR-1 and 28 RR-2 CPs in the pupal cuticle showed that the RR-1 CPs only existed in the endocuticle. A total of 19 RR-2 CPs existed in both the exocuticle and endocuticle and the other nine RR-2 CPs only occur in the exocuticle [11]. In the migratory locust *Locusta migratoria*, the DOMON domain protein LmKnk is localized in the newly formed adult cuticle by immunolocalization [12]. However, the structure and protein components of the cuticles during different developmental stages of holometabolous insects are unclear, especially in Lepidoptera, which are among the most severe agricultural pests. Moreover, there is not much clarity about protein components in the cuticles of different layers, especially the epicuticle.

Revealing the complex mechanism underlying cuticle formation will help us understand the molecular basis of the environmental adaptability in insects and provide a reference for pest control strategies. *B*. *mori* is holometabolous with a high-quality genome assembly and is a common experimental lepidopteran model. We used *B*. *mori* as a model to study cuticle formation in different developmental stages and layers. Serial sectioning and structure analysis of the cuticles during larval, pupal, and adult cuticle formations were conducted using frozen sections and immunofluorescence. This technique enabled us to determine the deposition periods of epicuticle, exocuticle, and endocuticle in the larva, pupa, and adult. Nine time points were selected for transcriptome sampling and analysis. Based on the expression and functional annotation analyses of the transcriptome, we identified the most probable structural protein genes involved in cuticle formations of the different developmental stages and layers. *BmorCPAP1-H*, a CP gene expressed in all stages, was selected for functional analysis by CRISPR/Cas9-mediated gene editing.

## 2. Results

### 2.1. Microstructures and Formation Processes of Larval, Pupal, and Adult Cuticles in B. mori

**Figure 1:**
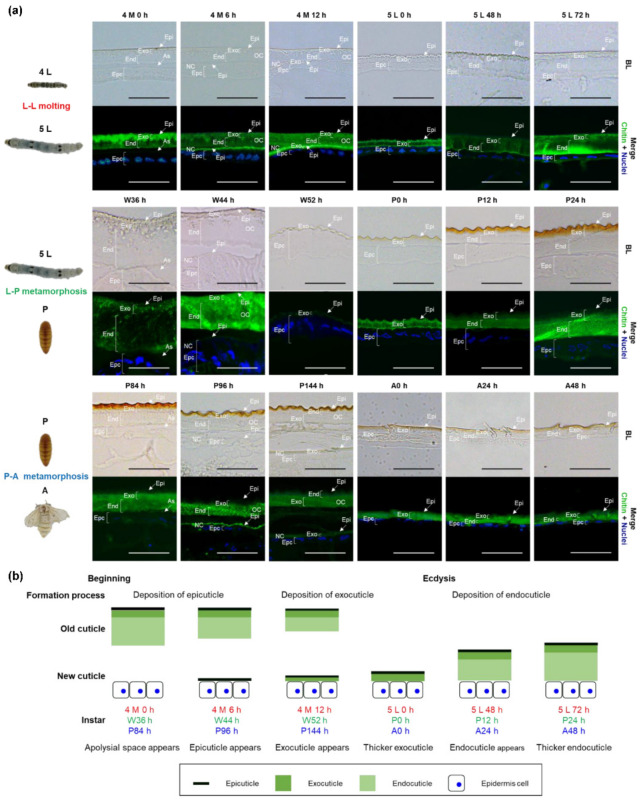
Microstructures and formation processes of larval, pupal, and adult cuticles in *Bombyx mori*. (**a**) Cuticle microstructures at representative time points. L-L molting: larval molting occurred from 4th instar larva (4 L) to 5th instar larva (5 L); L-P metamorphosis: larval metamorphosis occurred from 5 L to pupa (P); P-A metamorphosis: pupal metamorphosis occurred from P to adult (A); 4 M 0 h, 6 h, 12 h: 4th instar molting 0 h, 6 h, 12 h; 5 L 0 h, 48 h, 72 h; 5th instar larva 0 h, 48 h, 72 h; W36 h, 44 h, 52 h: wandering 36 h, 44 h, 52 h; P0 h, 12 h, 24 h, 84 h, 96 h, 144 h: pupa 0 h, 12 h, 24 h, 84 h, 96 h, 144 h; A0 h, 24 h, 48 h: adult 0 h, 24 h, 48 h; As: apolysis space; OC: old cuticle; NC: new cuticle; Epi: epicuticle; Exo: exocuticle; End: endocuticle; Epc: epidermal cells; Chitin: green; Nuclei: blue; Scale bar = 50 μm. (**b**) Schematic representation of the formation processes of larval, pupal, and adult cuticles.

From the 4th instar larva 72 h (4 L 72 h) to adult 72 h (A72 h), *B*. *mori* went through three developmental transitions. Larval molting (L-L molting) occurred from the 4th instar larva (4 L) to the 5th instar larva (5 L), larval metamorphosis (L-P metamorphosis) occurred from 5 L to pupa (P), and pupal metamorphosis (P-A metamorphosis) occurred from P to adult (A). The microstructures and formation processes of larval, pupal, and adult cuticles during these transitions were obtained from serial sectioning and characterized by cuticular structure by microscopy (Figure 1 and Figure S1). The apolysial space created by the separation of the epidermal cells from the old cuticle was observed in the 4th instar molting 0 h (4 M 0 h), wandering 36 h (W36 h), and pupa 84 h (P84 h), and new cuticle was found soon after these periods. In 4 M 6 h, W44 h, and P96 h, a very thin layer that was predicted to form the new epicuticle were observed over the upper face of the epidermal cells. In 4 M 12 h, W52 h, and P144 h, a thicker layer appeared and was supposed to include the epicuticle and some exocuticle. The exocuticle became thicker at the 5th instar larva 0 h (5 L 0 h), P0 h, and A0 h. The endocuticle was observed after ecdysis and thickened from 5 L 48 h to 5 L 72 h in the L-L molting stage and from P12 h to P24 h in the L-P metamorphosis stage. The endocuticle was present as a very thin layer with no obvious changes from A24 h to A72 h in the P-A metamorphosis stage. These results indicated that the epicuticle and exocuticle were deposited before ecdysis while the endocuticle was deposited after ecdysis during the cuticle formations of the larva, pupa, and adult (Figure 1b). A new epicuticle was first deposited during the early stages of each molt and metamorphosis. During the middle stages of each molt and metamorphosis, a new exocuticle appeared and became thicker until ecdysis was completed. After ecdysis, a new endocuticle appeared and became thicker.

### 2.2. Transcriptome Analysis during Larval, Pupal, and Adult Cuticle Formations in B. mori

**Figure 2:**
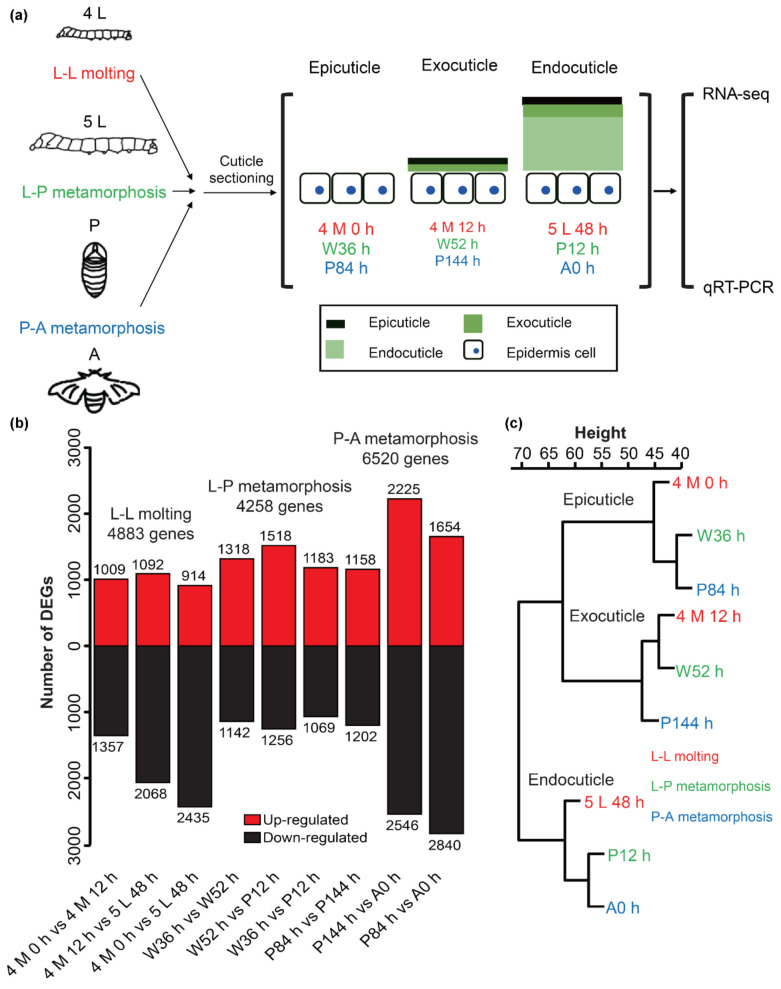
Sample collection and analysis of transcriptomes in *B*. *mori.* (**a**) Sample collection of transcriptomes. The epicuticle, exocuticle, and endocuticle in the top represented the developmental stages associated with epicuticle, exocuticle, and endocuticle depositions, respectively. qRT-PCR: real-time fluorescent quantitative PCR. (**b**) Statistics of differentially expressed genes (DEGs). (**c**) Cluster dendrogram showing a global relationship between different samples. Hierarchical cluster analyses of nine samples was performed based on the expression levels of DEGs. The *x*-axis is the degree of variance.

Understanding the structure and formation process of the cuticle could assist in accurate sampling for the transcriptome. Therefore, we selected nine time points before each layer thickened for the transcriptome sampling. The genes expressed in these periods might play an important role in cuticle formations of different developmental stages and layers (developmental stages associated with epicuticle formation: larva, 4 M 0 h; pupa, W36 h; adult, P84 h; developmental stages associated with exocuticle formation: larva, 4 M 12 h; pupa, W52 h; adult, P144 h; developmental stages associated with endocuticle formation: larva, 5 L 48 h; pupa, P12 h; adult, A0 h) (Figure 2a). The clean reads obtained from the nine libraries ranged from 46,599,108 to 65,190,354, and the clean bases ranged from 6.95 G to 9.65 G. Phred-like quality scores of the samples at the Q30 level ranged from 91.69% to 93.57% and the GC contents of the samples varied from 44.12% to 47.92% (Table S1). All the test results met the quality requirements and all the downstream analyses were based on the clean reads. Over 89.22% of the clean reads were mapped to the reference genome for each library (Table S2). By gene annotation analysis, 18,687 genes were detected in the transcriptome, of which 16,880 were matched to the reference genome, and the other 1807 were newly annotated genes. The gene expression levels of 18,687 genes in each library were counted by fragments per kilobase of exon per million reads (FPKM) and 12,748 genes were expressed in at least one sample with FPKM greater than 1 (Table S3).

Differential expression analyses were conducted for L-L molting, L-P metamorphosis, and P-A metamorphosis stages, respectively. For each stage, differentially expressed genes (DEGs) were identified between any two time points. Subsequently, 4883, 4258, and 6520 DEGs were identified in each stage (Figure 2b and Table S4). The expression analysis of the randomly selected 12 genes using real-time fluorescent quantitative PCR (qRT-PCR) indicated that the transcriptome and qRT-PCR data had high consistency with all correlation values greater than 0.95 (Figure S2). To examine the correlation of gene expression patterns among the nine samples, hierarchical cluster analysis was performed based on the expression levels of DEGs. The cluster dendrogram showed that the nine samples could be classified into three distinct groups: (1) 4 M 0 h, W36 h, and P84 h; (2) 4 M 12 h, W52 h, and P144 h; 3) 5 L 48 h, P12 h and A0 h (Figure 2c). These results revealed that three time points in each group shared similar expression patterns and they support our assertion, obtained by structure analyses of cuticle sections, that three time points in each group might be associated with epicuticle, exocuticle, or endocuticle formation.

### 2.3. CPs associated with Cuticle Formation in B. mori

**Figure 3:**
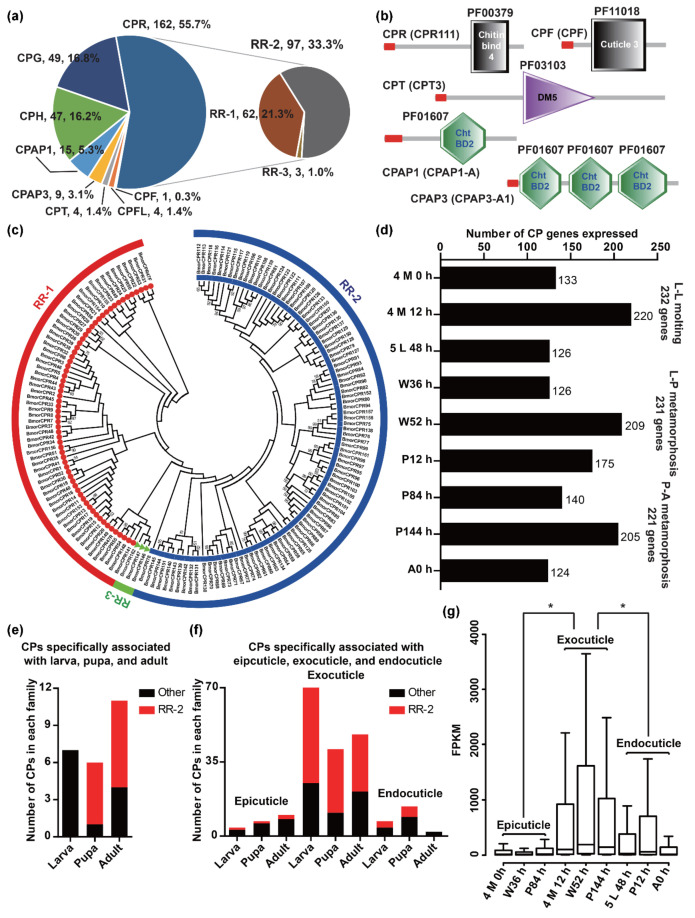
Identification, classification, and expression analyses of CPs in *B. mori*. (**a**) The distribution of CPs in each family. (**b**) Pfam domains of CPs in *B*. *mori*. The red rectangle represents the signal peptide. The CPs in CPR, CPF, CPT, and CPAP1 and CPAP3 protein families contain the domains of Chitin bind 4 (PF00379), Cuticle 3 (PF11018), DM5 (PF03103), and Cht BD2 (PF01607), respectively. (**c**) Phylogenetic relationships of CPs in the CPR protein family. A total of 162 CPs of the CPR protein family were classified into three subfamilies, 62 RR-1 (red circles), 97 RR-2 (blue squares), and three RR-3 (green triangle). (**d**) The number of CP genes expressed in our transcriptome. (**e**) The number of CPs specifically associated with larval, pupal, and adult cuticles. The red and black columns represent the CP families of RR-2 and others. (**f**) The number of CPs specifically associated with epicuticle, exocuticle, and endocuticle. The red and black columns represent the CP families of RR-2 and others. (**g**) The expression level of CP genes expressed in each developmental stage. The *p*-value was calculated by the Wilcoxon rank sum test and less than 0.05 was considered statistically significant. The asterisk indicated a significant difference between the two groups.

CPs are the most abundant component of the cuticle [13]. To study cuticle formation, it is necessary to identify CPs in the latest genome database of *B*. *mori*. A total of 291 CPs were identified and detailed information on these was obtained. Among the CPs, 31 were newly identified, the annotated gene models of 24% (69/291) were modified, and the coding sequences of 88% (255/291) were confirmed (Table S5). Based on the sequence features, 291 CPs were classified into eight families: 162 in CPR, 49 in CPG, 47 in CPH, 15 in CPAP1, nine in CPAP3, four in CPT, four in CPFL, and one in CPF (Figure 3a,b). Furthermore, 162 CPs of the CPR protein family were classified into three subfamilies with 62 RR-1, 97 RR-2, and three RR-3, by HMM and phylogenetic analysis (Figure 3a,c).

To identify the CP genes associated with cuticle formations of different developmental stages and layers, we conducted an expression analysis of CP genes in our transcriptome. Most CP genes (258/291) were expressed in at least one sample (Table S6). Among them, 232, 231, and 221 genes expressed in L-L molting, L-P metamorphosis, and P-A metamorphosis stages were associated with larval, pupal, and adult cuticle formations, respectively (Figure 3d). By comparing these CPs associated with larval, pupal, and adult, we found that 74% (192/258) were shared in larval, pupal, and adult (Figure S3), and seven, six, and eleven CPs were only in the larva, pupa, and adult, respectively (Figure 3e). Most CPs specifically associated with pupa (5/6) and adult (7/11) were RR-2. These results suggested that the CPs associated with larval, pupal, and adult cuticle formations were similar, and some CPs of the RR-2 protein family might play important roles in the cuticle formations of specific developmental stages.

The CP genes expressed in each developmental stage were associated with epicuticle, exocuticle, or endocuticle formation. We found that the number of CPs associated with exocuticle (4 M 12 h: 220; W52 h: 209; P144 h: 205) was larger than that of epicuticle (4 M 0 h: 133; W36 h: 126; P84 h: 140) and endocuticle (5 L 48 h: 126; P12 h: 175; A0 h: 124) (Figure 3d). By comparing these CPs associated with different layers, we found that most of the CPs (larva: 96; pupa: 110; adult: 87) associated with epicuticle and endocuticle formations were also in the exocuticle, and the majority of the CPs specifically associated with exocuticle formation were RR-2 (larva: 47/70; pupa: 30/41; adult: 27/48) (Figure 3f). In addition, the FPKM values of CP genes expressed in developmental stages associated with exocuticle formation (4 M 12 h, W52 h, and P144 h) were significantly higher than that of the epicuticle (4 M0 h, W36 h, and P84 h) and endocuticle (5 L 48 h, P12 h, and A0 h) (Figure 3g). Compared with the epicuticle and endocuticle, there were more CP genes involved in exocuticle formation, and these genes were also expressed at higher levels.

**Figure 4:**
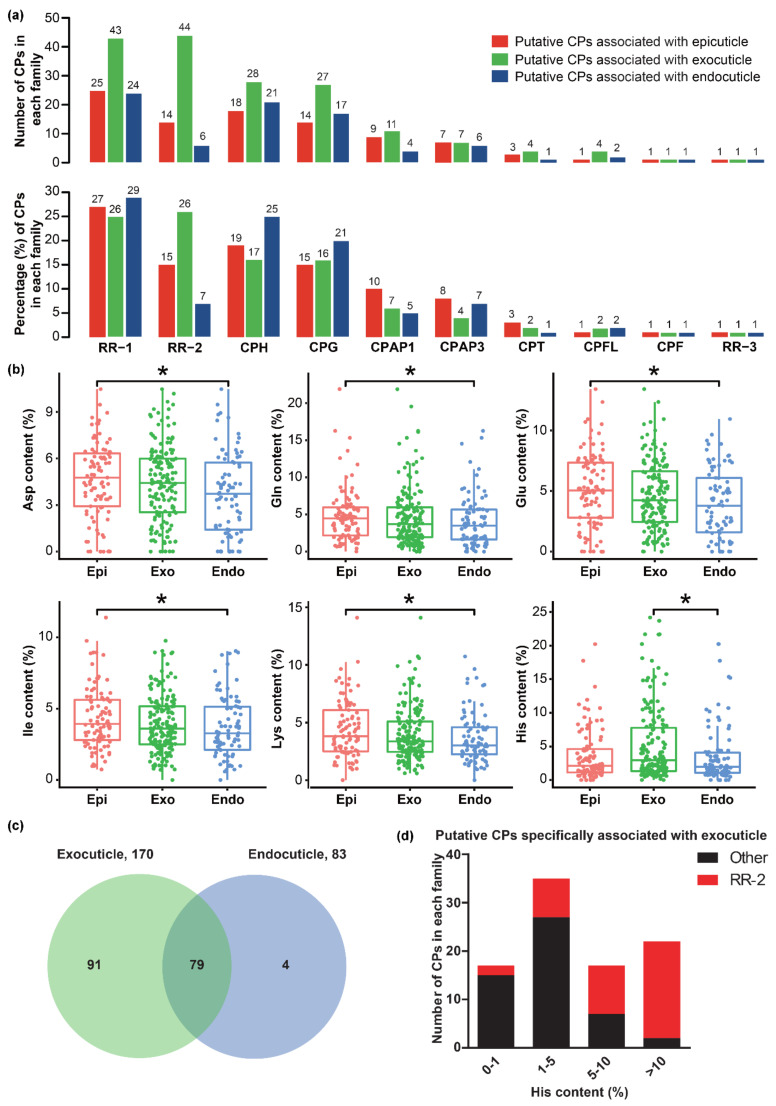
The putative CPs associated with epicuticle, exocuticle, and endocuticle in *B*. *mori*. (**a**) The number and percentage of putative CPs in each protein family. The red, green, and blue columns represent the putative CPs associated with epicuticle, exocuticle, and endocuticle, respectively. (**b**) Comparison of the amino acid contents among putative CPs associated with epicuticle, exocuticle, and endocuticle. The *p*-value was calculated by the Wilcoxon rank sum test and less than 0.05 was considered statistically significant. The asterisk indicated a significant difference between the two groups. Asp: aspartic acid; Gln: glutamine; Glu: glutamic acid; Ile: i-isoleucine; Lys: lysine; His: histidine. (**c**) Comparison of putative CPs associated with exocuticle and endocuticle; (**d**) The number of putative CPs specifically associated with exocuticle in each His content (%). The red and black column represents the CP families of RR-2 and others.

By comparing the CPs associated with the same layer in larval, pupal, and adult, we found that 93, 170, and 83 were shared in larval, pupal, and adult epicuticle, exocuticle, and endocuticle, respectively (Figure S3), and these CPs were regarded as putative CPs associated with epicuticle (93), exocuticle (170), and endocuticle (83). Gene family classification of these putative CPs showed that 10 CP families in *B*. *mori* might all take part in the formations of larval, pupal, and adult epicuticle, exocuticle, and endocuticle, especially the protein families of RR-1, RR-2, CPH, and CPG (Figure 4a). CPs of the RR-2 protein family are known to be expressed before ecdysis and are involved in constructing the exocuticle. We found that the numbers and percentages of putative CPs associated with exocuticle (44/170, 26%) in the RR-2 protein family were larger than that in the epicuticle (14/93, 15%) and endocuticle (6/83, 7%) (Figure 4a).

Amino acid compositions of CPs influence the physical properties of each layer, and therefore, we calculated and compared the amino acid content of putative CPs associated with epicuticle, exocuticle, and endocuticle. The contents of five kinds of amino acid (aspartic acid, Asp; glutamine, Gln; glutamic acid, Glu; i-isoleucine, Ile; lysine, Lys) and histidine (His) in putative CPs associated with epicuticle and exocuticle were significantly higher than in the endocuticle, respectively (Figure 4b). To determine which lead to a significant difference in His content between putative CPs associated with exocuticle and endocuticle, we compared these CPs. The results showed that most of the putative CPs associated with endocuticle (79/83) were shared in exocuticle, and 4 and 91 CPs were specifically associated with endocuticle and exocuticle, respectively (Figure 4c). Among 91 putative CPs specifically associated with exocuticle, the His content of 17 (2 RR-2 and 15 other families), 35 (8 RR-2 and 27 other families) 17 (10 RR-2 and 7 other families), and 22 (20 RR-2 and 2 other protein families) was 0%–1%, 1%–5%, 5%–10%, and >10%, respectively (Figure 4d). Therefore, the putative CPs specifically associated with exocuticle and contained high His contents might be the key determinations.

### 2.4. Other protein Components Involved in Cuticle Formation in B. mori

**Figure 5:**
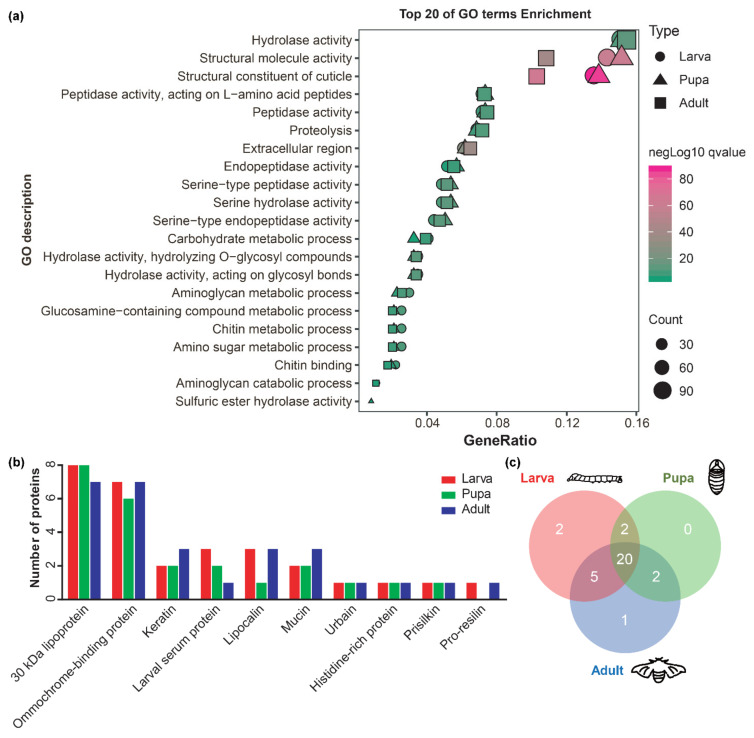
Identification of other protein components involved in larval, pupal, and adult cuticle formations in *B*. *mori*. (**a**) GO enrichment analysis of putative proteins involved in larval, pupal, and adult cuticle formations. (**b**) The number of the most probable other proteins in each protein family involved in larval, pupal, and adult cuticle formations. The red, green, and blue columns represent the most probable other proteins involved in larval, pupal, and adult cuticle formations. (**c**) Comparison of the most probable other proteins involved in larval, pupal, and adult cuticle formations.

A total of 649, 614, and 739 proteins with a signal peptide but without a transmembrane domain in L-L molting, L-P metamorphosis, and P-A metamorphosis stages might be involved in larval, pupal, and adult cuticle formations, respectively (Figure S4). GO enrichment analysis showed that these proteins tended to share GO terms (Figure 5a and Table S7). Based on functional annotation analysis, 29, 24, and 28 proteins were considered to be the most probable other proteins involved in larval, pupal, and adult cuticle formations (Table S8), and they could be classified into 10 functional categories by homology (Figure 5b). Among the most probable proteins, 20 proteins were shared by larval, pupal, and adult cuticle formations (Figure 5c).

There were at least 428 proteins in each stage with a signal peptide and FPKM >1 but without a transmembrane domain, which might be involved in the cuticle formation of each layer (Figure S5). GO enrichment analysis showed that the proteins involved in cuticle formations of different layers also tended to share GO terms. Specifically, in GO terms of a structural constituent of cuticle and structural molecular activity, their percentages in the exocuticle were higher than in the epicuticle and endocuticle (Figure S5 and Table S7). After filtering by functional annotation, at least 20 proteins were considered the most probable proteins involved in epicuticle, exocuticle, and endocuticle depositions of the larva, pupa, and adult (Table S9). Among them, most of the proteins (larva: 16, pupa: 19, adult: 17) were shared by the epicuticle, exocuticle, and endocuticle formations, and one histidine-rich protein only existed in the exocuticle formation.

### 2.5. BmorCPAP1-H Is Involved in Cuticle Formation in B. mori

**Figure 6:**
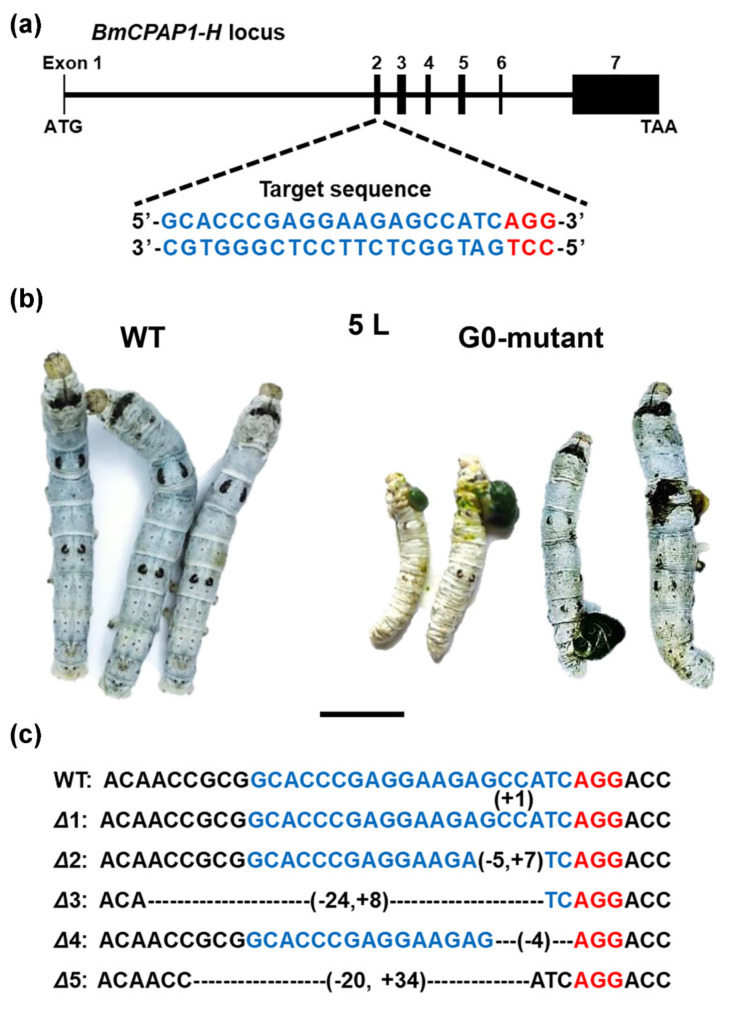
Disruption of *BmorCPAP1-H* using CRISPR/Cas9 in *B*. *mori*. (**a**) Genomic organization of *BmorCPAP1-H* ORF. The blue and red fonts indicate the target sequence and protospacer adjacent motif, respectively. CDS: coding sequence. (**b**) The phenotype of disruption of *BmorCPAP1-H*. In G0 generation, compared with wild-type (WT), the mutants exhibit the broken cuticle in the 5th instar larva (5 L). Bar, 1 cm. (**c**) Mutation site and recovered *BmorCPAP1-H* sequences in G0 mutants. The WT sequence is shown above the mutant sequences that contain deletions (-) and substitutions (+).

The CPs in the CPAP1 protein family contain one chitin-binding domain (ChtBD2 domain, PF01607), and have been reported to be involved in cuticle formation in the brown planthopper *Nilaparvata lugens* and the red flour beetle *Tribolium castaneum* [13,14]. However, there have been no functional studies on the CPAP1 protein family in *B*. *mori*. In this study, *BmorCPAP1-H* (*KWMTBOMO00699* and *00700*), expressed in all nine samples (Figure S6a), was selected for functional analysis. The temporal expression pattern showed that *BmorCPAP1-H* was expressed in all 26 time points from 4 L0 h to A24 h and highly expressed during each molt and metamorphosis, especially in 4 M (Figure S6b). The tissue expression pattern in 4 M showed that *BmorCPAP1-H* was expressed in all 12 tissues (head, silk gland, ventral nerve cord, epidermis cells, malpighia tubule, midgut, trachea, gonad, fat body, hemocyte, wing discs, and muscle) and highly expressed in the ventral nerve cord and epidermis. These results were consistent with the transcriptome data and suggested that BmorCPAP1-H might be involved in cuticle formation. To verify this inference, we disrupted *BmorCPAP1-H* using CRISPR/Cas9-mediated gene editing. After injection, a total of 78 eggs successfully hatched into larvae, and 26 were reared into adults. The remaining 52 larvae gradually died due to the broken cuticle, compared to the wild-type (WT), especially in 5 L (Figure 6b). Genomic sequencing of mutant individuals identified five kinds of frameshift mutations, all disrupting the *BmorCPAP1-H* coding region (Figure 6c).

## 3. Discussion

The cuticles of larva, pupa, and adult in holometabolous insects exhibit variable appearances and properties. However, there have been no detailed studies on the comparison of the larva, pupa, and adult cuticle formations. In this study, we systematically compared the deposition processes and the gene expression during larval, pupal, and adult cuticle formations in *B*. *mori*. Based on our results, we confirmed that the formations of larval, pupal, and adult cuticles in *B*. *mori* had a high similarity, such as the cuticle microstructure, the deposition process, and the type of cuticle structural protein.

Cuticle structure analysis showed that the deposition processes of larval, pupal, and adult cuticles were similar. Parallel results have been reported in the cuticle formations of the larva and adult wing of *B*. *mori*, the embryo and adult wing of the fruit fly *Drosophila melanogaster*, the adult wing of *T*. *castaneum*, the larva of the larger canna leafroller moth *Calpodes ethlius*, and the adult of the boll weevil *Anthonomus grandis* [9,15,16,17,18,19,20]. Therefore, we confirmed that the cuticles in different development stages are constructed in a similar manner.

Gene family identification analysis found a total of 291 CPs in the genome database of *B*. *mori.* Compared with the previous studies, 71 CPs were newly identified [21]. Gene expression analysis showed that the formations of larval, pupal, and adult cuticles shared 74% (192/258) of expressed CP genes. Among them, 29 CP genes were mentioned in previous studies, which reported that 31 CP genes were expressed during pupal and adult wing cuticle formations [9]. The special structure of larval, pupal, and adult cuticles might be caused by stage-specific CP genes. Seven, six, and eleven CP genes were specifically expressed during larval, pupal, and adult cuticle formations, respectively. Among them, *BmorCPR152* (*KWMTBOMO13020*) was only expressed in the P-A metamorphosis stage and is thought to be involved in wing scale construction [22]. In brief, accompanied by significant morphological variations during larva-pupa-adult transformation, the cuticleformations of *B*. *mori* in different developmental stages shared the majority of expressed CP genes and was only characterized by a few stage-specific CP genes.

It seems that formations of epicuticle, exocuticle, and endocuticle all require hundreds of CP genes. Previous studies suggested that the epicuticle mainly contains sclerotized proteins and lipoproteins. Our results demonstrated that CP genes are also expressed in the stages of epicuticle deposition and therefore may be incorporated into the epicuticle. Among them, BmorCPT1 (KWMTBOMO00298) is localized in the larval epicuticle by immunolocalization [23]. In addition, two CPs, PCP19 and PCP21, are localized in the pupal epicuticle by immunolocalization in *D. melanogaster* [24]. Gene family classification analysis showed that the CP genes expressed in stages of the epicuticle, exocuticle, and endocuticle depositions all contained the members of each CP family. Therefore, we confirmed that the CPs of all families in *B*. *mori* might be involved in epicuticle, exocuticle, and endocuticle depositions. Whether CPs of the RR-2 family are involved in endocuticle deposition has been controversial. Shahin et al. speculated that RR-1 CPs construct both layers of the exocuticle and endocuticle, while RR-2 CPs construct only the exocuticle [9]. However, we found that the CP genes of the RR-2 gene family were also expressed in the stages of exocuticle deposition. Therefore, we confirmed that RR-2 CPs are likely vital for endocuticle deposition, and this was supported by studies on *A*. *gambiae* and *N. lugens*. In *A*. *gambiae*, immunolocalization showed that 19 CPs of the RR-2 family were in both the exocuticle and the endocuticle [11]. In *N*. *lugens*, knockdown of six CP genes of the RR-2 gene family in 5th instar nymphs resulted in a thinned exocuticle and endocuticle of adults [13]. The His contents of exocuticle-related CPs were also significantly higher than His contents of the endocuticle. This is because more RR-2 CPs with high His contents were specifically expressed in the exocuticle-related samples. Since His residues in CPs were previously reported to be used for cuticle sclerotization [1,25], our results supported the previous findings that the exocuticle is rigid and hardened while the endocuticle is soft and flexible.

Although little information is available for the protein components encoded by non-CP genes in the cuticle, we identified ten kinds of other protein families that might be involved in cuticle deposition. These included larval serum protein, prisilkin, 30 KDa lipoprotein, lipocalin, ommochrome-binding protein, keratin, mucin, urbain, histidine-rich protein, and pro-resilin. Among them, larval serum proteins had been reported to be a protein component of the moth *Manduca sexta* and the blowfly *Calliphora vicina* cuticles [26,27]. In addition, the oyster *Pinctada fucata* prisilkin-39 (ACJ06766.1) can bind with chitin [28], which indicated that *prisilkin*-*39* (*KWMTBOMO13099*) expressed in cuticle may also be capable of combining with chitin in the cuticle of *B*. *mori*. Although the roles of other non-CP genes in the cuticle have not yet been studied, our results implied that these non-CP proteins might be involved in cuticle deposition.

Functional studies showed that *BmorCPAP1*-*H* affected the cuticle formation in *B*. *mori*. These results were consistent with previous studies in *N*. *lugens* and *T*. *castaneum* [13,14]. Our results increased our knowledge about the functions of CPAP1 CPs in Lepidoptera.

## 4. Materials and Methods

### 4.1. Insects

The *B. mori* strain, Dazao, was obtained from the Silkworm Gene Bank in Southwest University (Chongqing, China) and reared on mulberry leaves under a 12:12 h (L:D) photoperiod at 25 °C and 75% relative humidity. Under these conditions, the 4th instar larva (4 L), 4th instar molting (4 M), 5th instar larva (5 L), wandering (W), and pupa (P) stages lasted around 72, 24, 144, 60, and 192 h, respectively.

### 4.2. Sectioning, Staining, and Cuticular Structure Analysis

To determine the structures and formation processes of cuticles in different developmental stages of *B. mori*, we prepared serial cuticle sections of 30 time points from the end of the 4th instar larva to adult eclosion (Table S10) and characterized the cuticular structure by microscopy. The dorsal cuticle of the 4th abdominal segment was selected as the representative for cuticle sectioning, staining, and cuticular structure analysis, which was performed according to the protocol described by Xiong et al. [10]. The chitin and nuclei were visualized with a fluorescein-conjugated chitin-binding probe (green) and DAPI (blue), respectively.

### 4.3. Sample Collection, Library Preparation, Sequencing, and Analysis of Transcriptome

Nine time points were selected for transcriptome sequencing and the cuticles in these periods were collected. The cuticles of five individuals were pooled as one tissue sample and a total of 27 samples (3 samples for each time point) were collected. Total RNA was extracted from the samples using TRIzol reagent (Invitrogen, Carlsbad, CA, USA) according to the manufacturer’s instructions. For each time point, equal quantities of RNA isolated from three samples were pooled as one sample for library preparation. Sequencing libraries were generated using NEBNext^®^ Ultra™ RNA Library Prep Kit for Illumina^®^ (NEB, Ipswich, MA 01938, USA) following the manufacturer’s recommendations, and index codes were added to attribute sequences to each sample. The library preparations were sequenced on an Illumina HiSeq platform and 150 bp paired-end reads were generated.

Raw reads were first processed through fastp, and the clean reads were obtained in this step [29]. The latest genome data of *B*. *mori* in SilkBase (http://silkbase.ab.a.u-tokyo.ac.jp/cgi-bin/index.cgi, accessed on 7 February 2020) was used as the reference genome. The analysis of transcriptome was performed according to the protocol described by Kim et al., Trapnell et al., and Anders et al. [30,31,32]. The log2 (fold change) > 1 and false discovery rate (FDR) < 0.001 were set as the thresholds for significantly differential expression.

### 4.4. qRT-PCR Confirmation of Transcriptome Data

The 27 samples (3 samples for each time point) used in the transcriptome sequencing were also used for qRT-PCR. We randomly selected 12 genes that were differentially expressed according to transcriptomic analyses to verify the change in expression via qRT-PCR. The gene-specific primers of the randomly selected 12 genes are listed in Table S11. qRT-PCR assays were performed according to the protocol described by Shahin et al. [9]. The gene of *B*. *mori eukaryotic translation initiation factor 4A* (*KWMTBOMO02081*) was used as internal control and the relative expression was calculated using the 2^−ΔCt^ method.

### 4.5. Identification, Classification, Analysis of CPs in B. mori

All reported CPs were used as query sequences and the identification was performed according to the protocol described by Yan et al. [33]. All coding sequences of CPs were verified by blastn to search against the EST database in NCBI (http://www.ncbi.nlm.nih.gov, accessed on 10 September 2020) and full-length transcriptome data in our laboratory [34] with an E-value of 1 × 10^−5^. All identified CPs could be divided into different families based on the sequence features. The CPs of the CPR protein family were further classified by cuticleDB (http://bioinformatics.biol.uoa.gr/cuticleDB/, accessed on 13 September 2020) and phylogenetic analysis. The phylogenetic tree of CPs from the CPR protein family was constructed using MEGA5 with the neighbor-joining (NJ) method, with the bootstrap value set as 1000. The amino acid content of CPs was calculated using BioEdit. The Wilcoxon rank sum test was performed using the OmicShare tools, an online platform for data analysis (http://www.omicshare.com/tools, accessed on 2 May 2021).

### 4.6. Identification of Other Protein Components Associated with Cuticle Formation

Since the cuticle is an extracellular structure secreted by epidermal cells, the protein components in the cuticle should be secreted into extracellular areas [35]. Therefore, we speculated that proteins with a signal peptide but without a transmembrane domain were more likely involved in cuticle formation. Based on this possibility, we made a flow chart for the identification of other protein components involved in cuticle formation (Figure S7). The protein sequences encoded by DEGs in each stage were used to predict signal peptide and transmembrane domains, respectively. Based on predicted results, we obtained the proteins with a signal peptide but without a transmembrane domain, which were considered the putative proteins involved in cuticle formation. Among them, the proteins in the L-L molting, L-P metamorphosis, and P-A metamorphosis stages might be involved in larval, pupal, and adult cuticle formation, respectively, and the proteins in each sample with FPKM >1 might be involved in epicuticle, exocuticle, or endocuticle formation. All of these putative proteins were used to perform functional annotation by several methods, including GO annotation, Pfam, Interpro, blast to nr, and blast to FlyBase (http://flybase.org/, accessed on 6 July 2021). The combination of these functional annotation results identified the most probable proteins involved in cuticle formation of the different developmental stages and layers.

### 4.7. Spatiotemporal Expression Pattern

*B*. *mori* samples at 26 time points from 4 L0 h to A24 h and 12 tissues (head, silk gland, ventral nerve cord, epidermis cells, malpighia tubule, midgut, trachea, gonad, fat body, hemocyte, wing discs, and muscle) in 4 M *B*. *mori* were collected for spatiotemporal expression pattern analysis. Gene-specific primers of *BmorCPAP1-H* are listed in Table S11. The method of quantification is the same as that in 4.4.

### 4.8. CRISPR/Cas9-Mediated Gene Knockout

The sgRNA was designed by CRISPRdirect (http://crispr.dbcls.jp/, accessed on 6 July 2021) and synthesized using the RiboMAXTM Large Scale RNA Production System T7 kit (Promega, Madison, USA). A single guide RNA (sgRNA) targeting a specific site was designed in the second coding sequence of *BmorCPAP1-H* (Figure 6a), and then we simultaneously injected the sgRNA and Cas9 proteins into newly laid embryos (G0 generation). To identify the mutation site and type of larvae edited by CRISPR/Cas9, we used the appropriate primer sets (F: AACCGTACAATGCCTAA; R: GGGTTTATACTAGATTCACAG) to clone and sequence the genomic DNA.

### 4.9. Other Analysis Tools

The hierarchical clustering of the samples, GO enrichment analysis, and the Wilcoxon rank sum test were performed using the OmicShare tools. InterProScan provided functional annotation analysis of proteins by classifying them into families and predicting domains and important sites, including the Pfam, Interpro, signal peptides, and transmembrane domains [36].

## 5. Conclusions

We obtained a blueprint of cuticle microstructures and stage-specific transcriptome dynamics during cuticle formation in the larva, pupa, and adult. Results showed that structural proteins associated with larval, pupal, and adult cuticles were similar and cuticular proteins were the major components. The study also provided a set of useful candidate genes for future investigation into molecular mechanisms underlying cuticle formation. Overall, the results have increased our understanding of the morphology and molecular basis of cuticle formation and may contribute to the development of insecticides targeting cuticle-related proteins.

## Data Availability

The raw transcriptome reads obtained during this study are available at NCBI BioProject under accession number PRJNA784028.

## Supplementary Materials

The following supporting information can be downloaded at: https://www.mdpi.com/article/10.3390/ijms23095155/s1.
