# Supplementary material for: A Blueprint of Microstructures and Stage-Specific Transcriptome Dynamics of Cuticle Formation in Bombyx mori
Source: Int J Mol Sci. 2022 May 5;23(9):5155. doi: 10.3390/ijms23095155 (PMC9101387; doi:10.3390/ijms23095155)
Supplement: Supplementary file 1 [file ijms-23-05155-s001.zip › Supplementary.pdf]

Supplementary figures

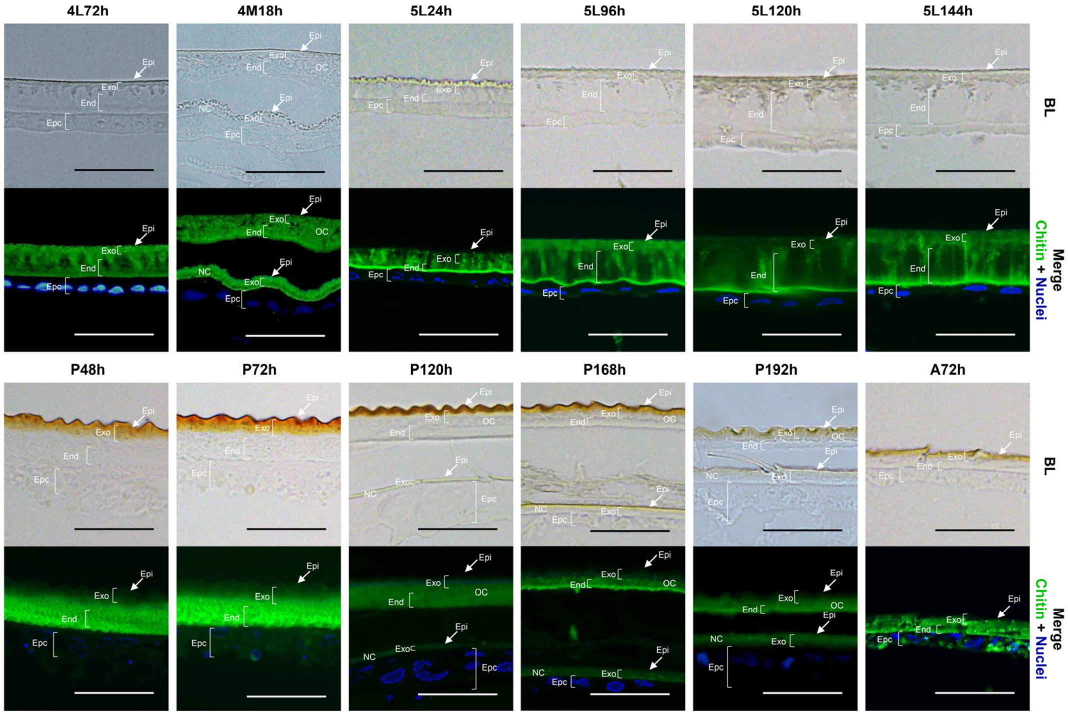

Figure S1. the microstructures of cuticle sections at various developmental stages in *Bombyx mori*. 4 L 72 h: 4th instar larva 72 h; 4 M 18 h: 4th instar molting 18 h; 5 L 24 h, 96 h, 120 h, 144 h: 5th instar larva 24 h, 96 h, 120 h, 144 h; P48 h, 72 h, 120 h, 168 h, 192 h: pupa 48 h, 72 h, 120 h, 168 h, 192 h; A72 h: adult 72 h; Chitin: green; Nuclei: blue; Scale bar = 50 μm.

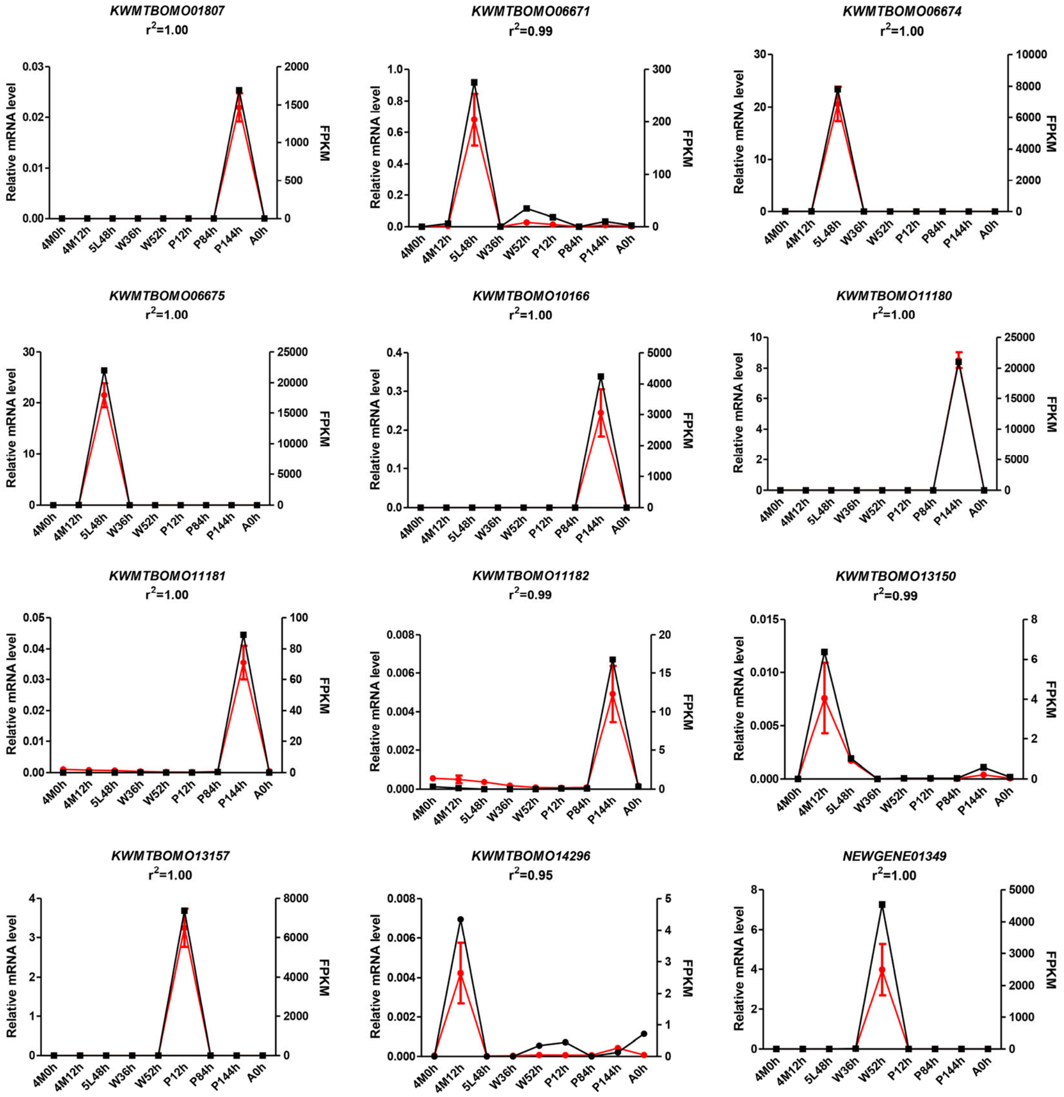

Figure S2. real-time fluorescent quantitative PCR (qRT-PCR**)** validation of transcriptome data. The left and right vertical axis indicated the relative mRNA level by qRT-PCR (read lines) and the vertical axis indicated the FPKM by transcriptome analysis (black lines). The horizontal axis in-dicated the developmental stages, respectively. The r^2 value showed correlation between the two methods.

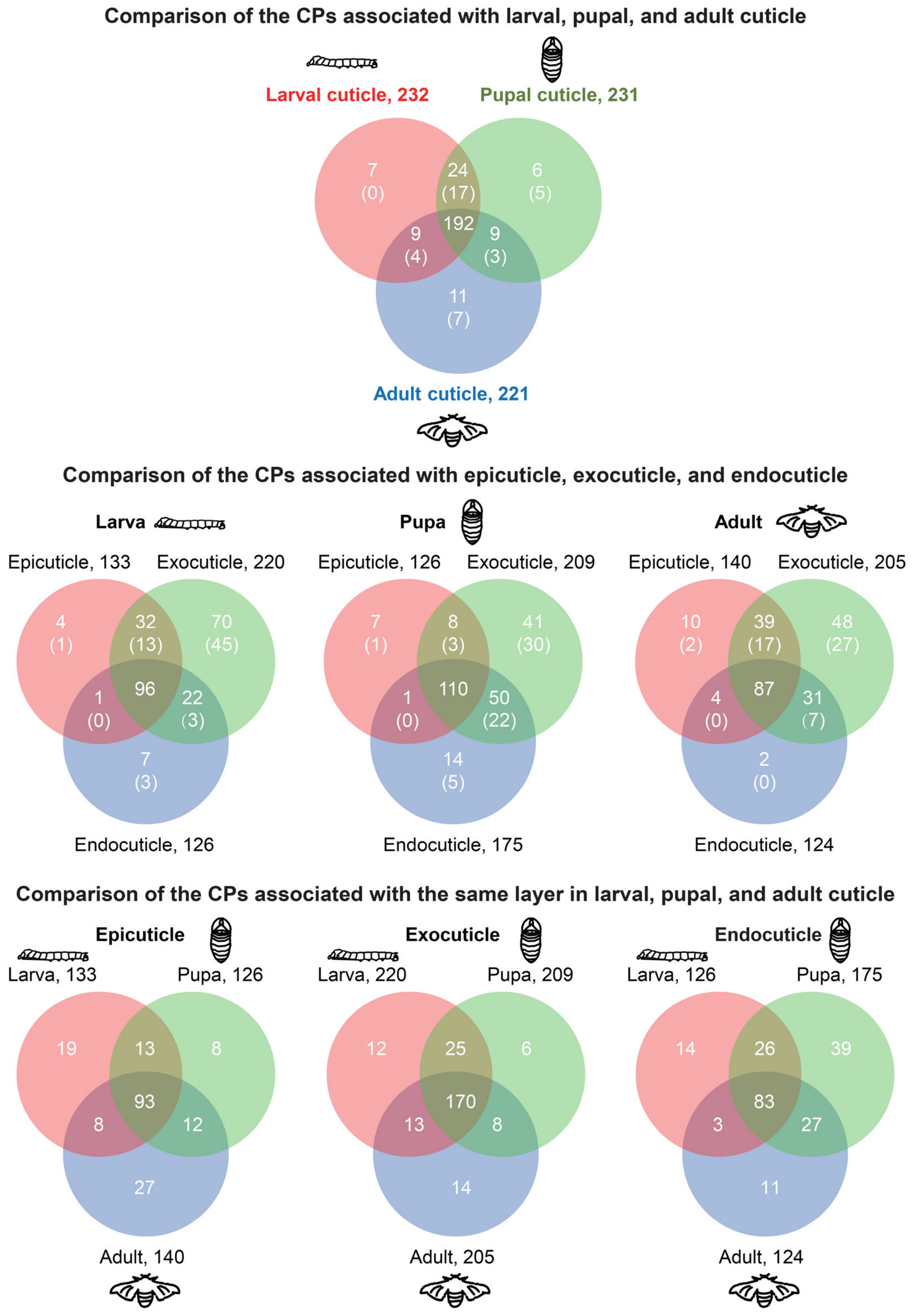

Figure S3. comparison of the CPs associated with cuticle formation. The numbers in brackets indicated the number of CPs from the RR-2 protein family.

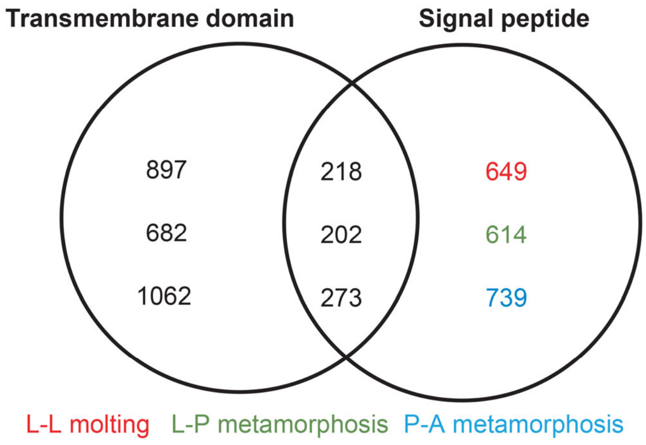

Figure S4. Venn diagrams showed that 649, 614, and 739 proteins with signal peptide but without transmembrane domain were obtained in L-L molting, L-P metamorphosis, and P-A metamorphosis stages, respectively.

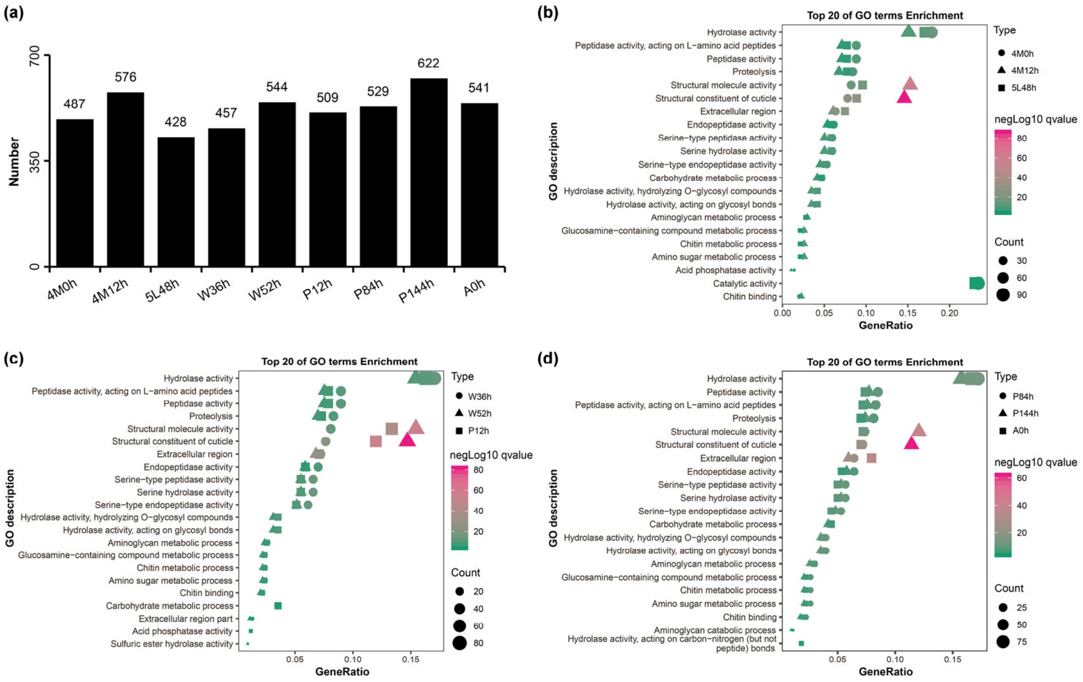

Figure S5. putative other proteins involved in cuticle formations of different layers. (**a**) The number of putative other proteins involved in cuticle formations of different layers. Epicuticle: 4 M 0 h, W36 h, and P84 h; Exocuticle: 4 M 12 h, W52 h, and P144 h; Endocuticle: 5 L 48 h, P12 h, and A0 h; (**b**-**d**) GO enrichment analysis of putative proteins involved in epicuticle, exocuticle, and endocuticle for-mations of larva (**b**), pupa (**c**), and adult (**d**).

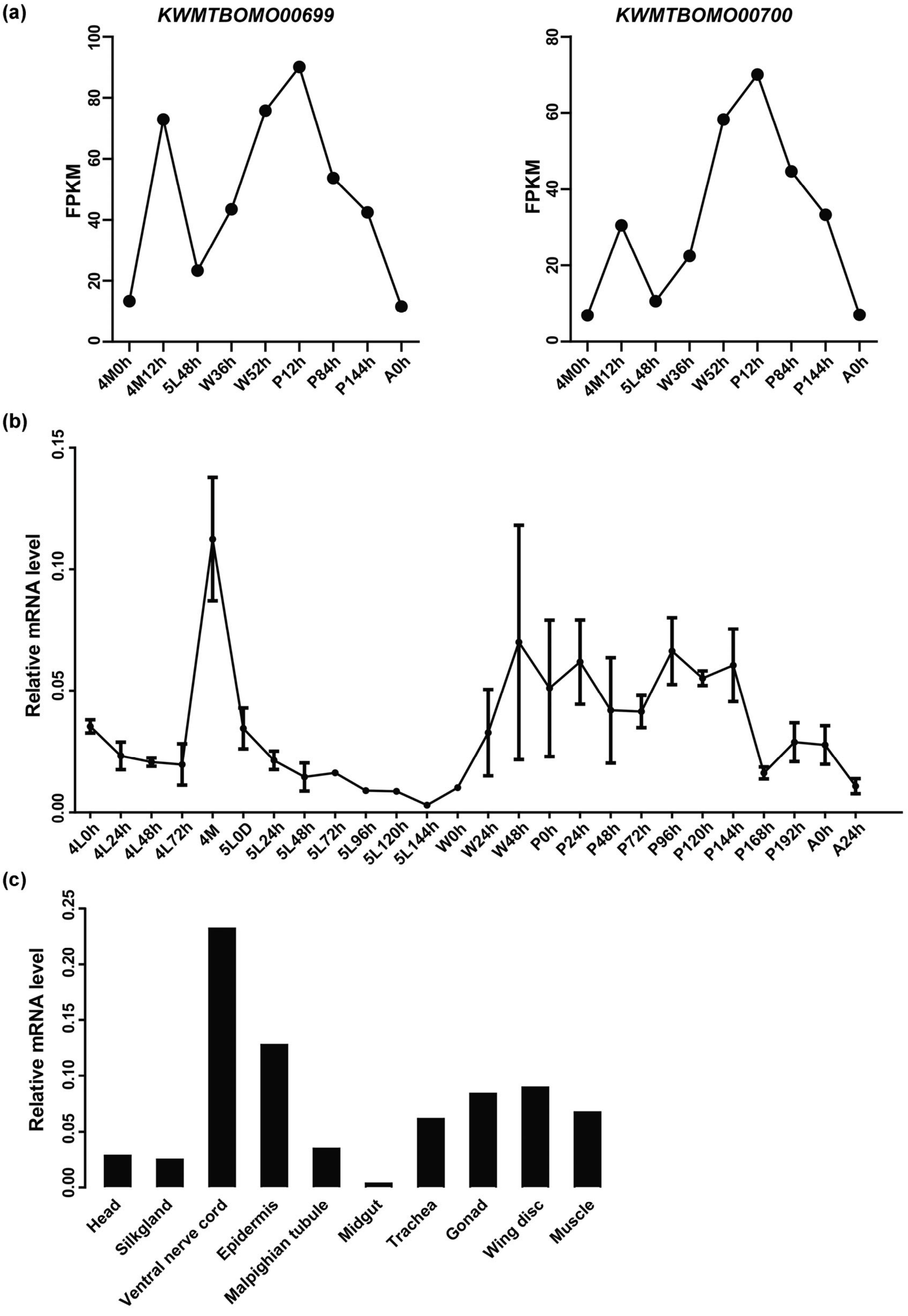

Figure S6. the spatiotemporal expression pattern of *BmorCPAP1-H* in *B. mori*. (**a**) Expression pattern of *BmorCPAP1-H* in transcriptome data. *Bmor-CPAP1-H* was annotated as *KWMTBOMO00699* and *00700* in genome data; (**b**) Temporal

expression pattern of *BmorCPAP1-H*; (**c**) Tissue expression pattern of *BmorCPAP1-H* in 4th instar molting (4M).

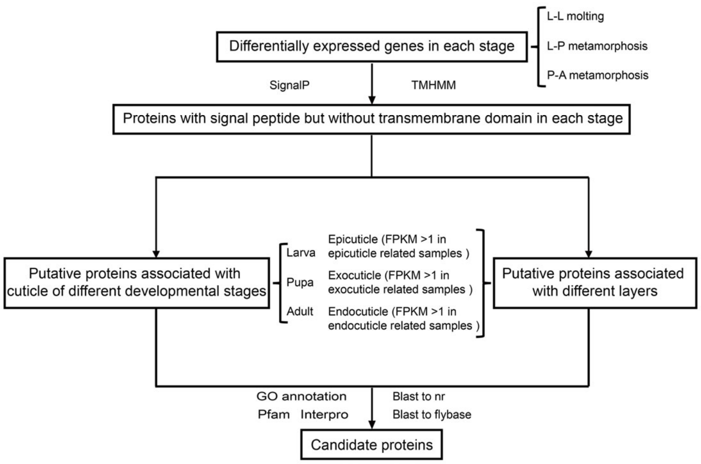

Figure S7. flow chart for identification of other protein components involved cuticle formation.

Supplementary tables:

Table S1: major characteristics of transcriptome data

Table S2: summary of transcriptome data mapped to reference genome

Table S3: FPKM values of genes in our transcriptome

Table S4: DEGs in each pairwise comparison

Table S5: the detailed information of cuticular proteins (CPs) in *B. mori*

Table S6: the expression analysis of CP genes

Table S7: GO enrichment analysis of putative proteins involved in cuticle formation

Table S8: functional annotation of putative other proteins involved in cuticle formation

Table S9: the most probable other protein components involved in cuticle formations of different layers

Table S10: the time points for cuticle sections

Table S11: list of qRT-PCR primers
