# Supplementary material for: A Blueprint of Microstructures and Stage-Specific Transcriptome Dynamics of Cuticle Formation in Bombyx mori
Source: Int J Mol Sci. 2022 May 5;23(9):5155. doi: 10.3390/ijms23095155 (PMC9101387; doi:10.3390/ijms23095155)
Supplement: Supplementary file 1 [file ijms-23-05155-s001.zip › Table S1.pdf]

**Table S1.** Major characteristics of transcriptome data

| Sample name | Raw reads | Clean reads | Clean bases | Q20 (%) | Q30 (%) | GC content (%) |
|---|---|---|---|---|---|---|
| 4 M 0 h | 63243156 | 60543396 | 8.97G | 97.20 | 92.24 | 45.44 |
| 4 M 12 h | 68366516 | 65190354 | 9.65G | 96.95 | 91.69 | 47.92 |
| 5 L 48 h | 66932280 | 64175614 | 9.51G | 97.19 | 92.21 | 46.60 |
| W36 h | 60619302 | 59749232 | 8.91G | 97.90 | 93.41 | 44.12 |
| W52 h | 47398616 | 46599108 | 6.95G | 97.90 | 93.46 | 46.06 |
| P12 h | 55603048 | 54836524 | 8.18G | 97.94 | 93.57 | 47.11 |
| P84 h | 52483986 | 51669148 | 7.71G | 97.87 | 93.35 | 44.31 |
| P144 h | 62894952 | 61895388 | 9.22G | 97.91 | 93.49 | 46.40 |
| A0 h | 58671436 | 57850566 | 8.59G | 97.84 | 93.30 | 46.09 |
