# Supplementary material for: A Blueprint of Microstructures and Stage-Specific Transcriptome Dynamics of Cuticle Formation in Bombyx mori
Source: Int J Mol Sci. 2022 May 5;23(9):5155. doi: 10.3390/ijms23095155 (PMC9101387; doi:10.3390/ijms23095155)
Supplement: Supplementary file 1 [file ijms-23-05155-s001.zip › Table S10.pdf]

Table S10. The time points for cuticle sections

Stages	Time points
4th instar larva (4 L)	4 L 72 h
4th instar molting (4 M)	4 M 0 h, 4 M 6 h, 4 M 12 h, 4 M 18h
5th instar larva (5 L)	5 L 0 h, 5 L 24 h, 5 L 48 h, 5 L 72 h, 5 L 96 h, 5 L 120 h, 5 L 144 h
Wandering (W)	W36 h, W44 h, W52 h
Pupa (P)	P0 h, P12 h, P24 h, P48 h, P72 h, P84 h, P96 h, P120 h, P144 h, P168 h, P192 h
Adult (A)	A0 h, A24 h, A48 h, A72 h
