# Supplementary material for: A Blueprint of Microstructures and Stage-Specific Transcriptome Dynamics of Cuticle Formation in Bombyx mori
Source: Int J Mol Sci. 2022 May 5;23(9):5155. doi: 10.3390/ijms23095155 (PMC9101387; doi:10.3390/ijms23095155)
Supplement: Supplementary file 1 [file ijms-23-05155-s001.zip › Table S11.pdf]

Table S11. List of qRT-PCR primers

Gene	Forward primer (5'-3')	Reverse primer (5'-3')
KWMTBOMO01807	CACCATCAGAGTAGTGGACTAC	CCTGTTTCGTTATAAGCGTCTG
KWMTBOMO06671	TCTCGCCTTCGCCACATT	AGCGTAGGACGACAGAGGGTAG
KWMTBOMO06674	CAACATATACTGCCCCGGTTTA	TGTACGCTGGGGCAGTATAT
KWMTBOMO06675	CATCCTACGTACCTTCCACCTA	CGTTGTAGTAGTTGGGGTATGA
KWMTBOMO10166	CTATAGCTTCTCGTACGGGGTC	GATCTTATCAGCGGTGTACGTA
KWMTBOMO11180	CGTCATTATACAAGGATCACGC	GGGACCAGATTTGATTATTGGC
KWMTBOMO11181	ACGCGATTGTCAAGAATATTGG	TTCCGTACCGAAACCGTAATTA
KWMTBOMO11182	GAGCTACAGTTTCAACTATGCG	GATTGACAGCGAATGCACATT
KWMTBOMO13150	CATCCAAGCTTCAAACCTCATC	TTTCTCGGTAACAGGTGATTGT
KWMTBOMO13157	GTATCAGACATGAGCAGGAA	TAACCAGCCATAGAACCC
KWMTBOMO14296	GGTTATTCAGTGAAAGACGCTC	GTATTCCCTCCTCGGTTGTAAT
NEWGENE01349	GAGAGTATAGTCTTCACGAGGC	AGTCCGGAGTTGATAACTTCAG
BmorCPAP1-H	AACCGTACAATGCCTAA	GGGTTTATACTAGATTCACAG
KWMTBOMO02081	TTCGTACTGCTCTTCTCGT	CAAAGTTGATAGCAATTCCCT
