# Supplementary material for: A Blueprint of Microstructures and Stage-Specific Transcriptome Dynamics of Cuticle Formation in Bombyx mori
Source: Int J Mol Sci. 2022 May 5;23(9):5155. doi: 10.3390/ijms23095155 (PMC9101387; doi:10.3390/ijms23095155)
Supplement: Supplementary file 1 [file ijms-23-05155-s001.zip › Table S2.pdf]

Table S2. Summary of transcriptome data mapped to silkworm reference genome

Sample name	4 M 0 h	4 M 12 h	5 L 48 h	W36 h	W52 h	P12 h	P84 h	P144 h	A0 h
Total reads	60543396	65190354	64175614	59749232	46599108	54836524	51669148	61895388	57850566
Total mapped	55347648 (91.42%)	58161932 (89.22%)	58041838 (90.44%)	56740930 (94.97%)	43917344 (94.25%)	51782670 (94.43%)	48548034 (93.96%)	58221274 (94.06%)	53472216 (92.43%)
Multiple mapped	3523216 (5.82%)	4138250 (6.35%)	4711886 (7.34%)	4246358 (7.11%)	4087722 (8.77%)	4817726 (8.79%)	4499070 (8.71%)	5986736 (9.67%)	5366984 (9.28%)
Uniquely mapped	51824432 (85.60%)	54023682 (82.87%)	53329952 (83.10%)	52494572 (87.86%)	39829622 (85.47%)	46964944 (85.65%)	44048964 (85.25%)	52234538 (84.39%)	48105232 (83.15%)
