# Supplementary material for: A Blueprint of Microstructures and Stage-Specific Transcriptome Dynamics of Cuticle Formation in Bombyx mori
Source: Int J Mol Sci. 2022 May 5;23(9):5155. doi: 10.3390/ijms23095155 (PMC9101387; doi:10.3390/ijms23095155)
Supplement: Supplementary file 1 [file ijms-23-05155-s001.zip › Table S9.pdf]

Table S9. The most probable other protein components involved in cuticle depositions of different layers in *B. mori*.

Functional classification	Larva			Pupa			Adult		
	Epi (4 M 0 h)	Exo (4 M 12 h)	End (5 L 48 h)	Epi (W36 h)	Exo (W52 h)	End (P12 h)	Epi (P84 h)	Exo (P144 h)	End (A0 h)
30 kDa lipoprotein	6	7	8	7	8	8	6	6	7
Ommochrome-binding protein	5	5	7	5	6	5	6	5	6
Keratin	0	2	2	1	2	2	3	3	3
Larval serum protein	3	3	3	2	1	2	1	1	0
Lipocalin	3	3	2	1	1	1	3	3	2
Mucin	2	1	0	2	2	2	3	2	1
Urbain	1	1	0	1	1	1	1	0	0
Histidine-rich protein	0	1	0	0	1	0	0	1	0
Prisilkin	0	1	0	1	1	1	1	1	1
Pro-resilin	0	1	0	0	0	0	0	1	1
Total	20	25	22	20	23	22	24	23	21
